# Meiotic crossover reduction by virus‐induced gene silencing enables the efficient generation of chromosome substitution lines and reverse breeding in *Arabidopsis thaliana*

**DOI:** 10.1111/tpj.14990

**Published:** 2020-10-20

**Authors:** Vanesa Calvo‐Baltanás, Cris L. Wijnen, Chao Yang, Nina Lukhovitskaya, C. Bastiaan de Snoo, Linus Hohenwarter, Joost J. B. Keurentjes, Hans de Jong, Arp Schnittger, Erik Wijnker

**Affiliations:** ^1^ Laboratory of Genetics Wageningen University & Research Droevendaalsesteeg 1 Wageningen 6708 PB the Netherlands; ^2^ Department of Developmental Biology Institut für Pflanzenwissenschaften und Mikrobiologie University of Hamburg Ohnhorststrasse 18 Hamburg 22609 Germany; ^3^ Centre National de la Recherche Scientifique Institut de Biologie Moléculaire des Plantes Université de Strasbourg 12, rue du général Zimmer Strasbourg 67084 France; ^4^ Rijk Zwaan R&D Fijnaart Eerste Kruisweg 9 Fijnaart 4793 RS the Netherlands; ^5^ Present address: Department of Biological Sciences National University of Singapore 14 Science Drive 4 Singapore 117543 Singapore; ^6^ Present address: Division of Virology Department of Pathology University of Cambridge Tennis Court Rd Cambridge CB2 1QP UK

**Keywords:** meiosis, MSH5, virus‐induced gene silencing, chromosome substitution lines, reverse breeding, *Arabidopsis thaliana*, technical advance

## Abstract

Plant breeding applications exploiting meiotic mutant phenotypes (like the increase or decrease of crossover (CO) recombination) have been proposed over the last years. As recessive meiotic mutations in breeding lines may affect fertility or have other pleiotropic effects, transient silencing techniques may be preferred. Reverse breeding is a breeding technique that would benefit from the transient downregulation of CO formation. The technique is essentially the opposite of plant hybridization: a method to extract parental lines from a hybrid. The method can also be used to efficiently generate chromosome substitution lines (CSLs). For successful reverse breeding, the two homologous chromosome sets of a heterozygous plant must be divided over two haploid complements, which can be achieved by the suppression of meiotic CO recombination and the subsequent production of doubled haploid plants. Here we show the feasibility of transiently reducing CO formation using virus‐induced gene silencing (VIGS) by targeting the meiotic gene *MSH5* in a wild‐type heterozygote of *Arabidopsis thaliana*. The application of VIGS (rather than using lengthy stable transformation) generates transgene‐free offspring with the desired genetic composition: we obtained parental lines from a wild‐type heterozygous F_1_ in two generations. In addition, we obtained 20 (of the 32 possible) CSLs in one experiment. Our results demonstrate that meiosis can be modulated at will in *A. thaliana* to generate CSLs and parental lines rapidly for hybrid breeding. Furthermore, we illustrate how the modification of meiosis using VIGS can open routes to develop efficient plant breeding strategies.

## Significance Statement

Changing meiotic recombination rates can support plant research and breeding, but is rarely achieved in a transient way (i.e. where offspring fertility is not compromised). Here we show that virus‐induced gene silencing (VIGS) can be used to temporarily downregulate crossover recombination in *Arabidopsis thaliana* hybrids to facilitate the generation of chromosome substitution lines, reverse breeding and reverse breeding‐like applications.

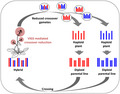

## INTRODUCTION

Fuelled by the description of a variety of meiotic mutants in plants, interest has grown for exploring the use of mutant meiotic phenotypes for improving plant breeding strategies (Wijnker and de Jong, 2008; D’Erfurth *et al*., 2009; Dirks *et al*., 2009; Wijnker *et al*., 2012; Mieulet *et al*., 2016, 2018; Lambing *et al*., 2017; Blary *et al*., 2018; Wang *et al*., 2019). For example, mutations causing the increase or decrease of recombination can be used to generate mapping populations that consist of either high‐recombinant or low‐recombinant offspring (Dirks *et al*., 2009; Crismani *et al*., 2012; Wijnker *et al*., 2012, 2014; Séguéla‐Arnaud *et al*., 2017; Fernandes *et al*., 2018; Mieulet *et al*., 2018; Wijnen *et al*., 2018). As mutations that alter meiotic recombination rates can adversely affect fertility (i.e. through defective DNA repair or the mis‐segregation of chromosomes) or may have pleiotropic effects, it would be highly practical to be able to transiently change recombination rates in specific plants. Here, we describe the use of virus‐induced gene silencing (VIGS), a transient silencing technique, to reduce crossover (CO) formation. This serves to illustrate the feasibility of directing the genetic composition of offspring, but also explores its use in possible breeding applications.

The interest in CO suppression for basic and applied research lies in the possibility to generate chromosome substitution lines (CSLs) quickly, in which one or more chromosomes of one parent are introgressed into the background of another parent. CSLs are outstanding tools for mapping quantitative trait loci (QTLs) in mice and *Arabidopsis thaliana*, where they have been used to map recombination modifiers (Nadeau *et al*., 2000; Singer *et al*., 2004; Spiezio *et al*., 2012; Ziolkowski *et al*., 2017). In addition, the generation of a complete CSL population in *A. thaliana* allowed the systematic detection of two‐way and three‐way epistatic (non‐additive) interactions for different traits (Wijnen *et al*., 2018).

**Figure 1:**
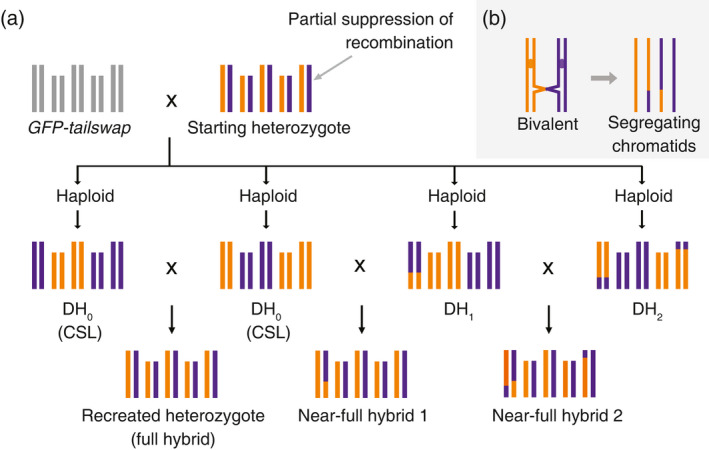
Reverse breeding (heterozygote reconstruction) through partial crossover (CO) suppression and doubled haploid (DH) production in *Arabidopsis thaliana*. (a) A starting *A. thaliana* heterozygote (top) is selected for which parental lines are to be made. Five chromosome pairs are shown, with homologs in orange and purple. Meiotic COs are partially suppressed in this heterozygote, after which pollen grains are used to pollinate the *A. thaliana* haploid inducer line *GFP‐tailswap* (Ravi and Chan, 2010) to generate haploid, and subsequently DH offspring. Example offspring are shown in the middle row, having 0, 1 or 2 COs (DH_0_, DH_1_ and DH_2_, respectively). Note that DH_0_ plants are also chromosome substitution lines (CSLs). Crossing complementing DH_0_ (left) recreates the heterozygote as a full hybrid (bottom row, left), an approach similar to that described by Wijnker *et al*. (2012). Crossing DH_0_ with DH_1_ (middle) or DH_1_ with DH_2_ (right) generates near‐full hybrids 1 and 2, which have small homozygous genomic regions. Note that in the cross of DH_1_ with a DH_2_, chromosome 1 is largely heterozygous, as the parental lines complement one another in the distal chromosome region. (b) Recombinant but also non‐recombinant chromatids segregate in the presence of a CO. Detail of a bivalent pair with one meiotic CO is shown (left). Only two of the four resulting chromatids are recombinant (right).

Traditionally, CSLs are generated by crossing an F_1_ hybrid with one of its parents, followed by several rounds of backcrossing, ultimately to select for offspring with specific non‐recombinant chromosomes (Nadeau *et al*., 2000; Koumproglou *et al*., 2002). This is a labour‐intensive process that is particularly protracted in species with long generation times. In plants, however, CSLs can be generated more efficiently when CO recombination is reduced or completely suppressed in the hybrid (Dirks *et al*., 2009; Wijnker *et al*., 2012). In the latter case, only non‐recombinant chromosomes segregate to gametes. Such haploid gametes can be used in backcrosses or can be grown directly into haploid offspring that carry different chromosome combinations of the parental lines. Haploid plants that are derived from gametes carrying non‐recombinant chromosomes can then give rise to diploid homozygous lines, known as doubled haploids (DHs), which are also CSLs (Figure 1a) (Dirks *et al*., 2009; Wijnker *et al*., 2012; Wijnen *et al*., 2018). Direct CO suppression in a hybrid coupled with DH technology can deliver CSLs in just two generations (Figure 1a).

The generation of CSLs may have further advantages. A breeding technique termed ‘reverse breeding’ exploits CSL generation from a heterozygous plant to obtain parental lines that can recreate the heterozygous genotype as an F_1_ hybrid (Link and Melchinger, 1995; Dirks *et al*., 2009; Wijnker *et al*., 2012). In the anticipated application of reverse breeding a favourable heterozygote is selected directly from an outcrossing population, after which its parental lines are generated. During reverse breeding, CSL offspring are generated from the selected heterozygote through CO suppression followed by DH production. Only a finite number of CSLs can be generated from a heterozygous plant and this number equals 2*^x^*, in which *x* is the haploid chromosome number of the species. In reverse breeding, CSLs are generated until a so‐called ‘complementing’ pair of lines is found: two CSLs that, when crossed, reconstitute the starting heterozygote (Figure 1a). Reverse breeding was shown to be practically feasible in a proof‐of‐concept study (Wijnker *et al*., 2012), but the approach presented two main drawbacks: complete CO suppression induced semi‐sterility and the downregulation of CO recombination in a plant required the use of a stable transgene. These points are addressed in more detail in the following sections.

### Partial versus complete CO suppression

The suppression of COs may come at a cost because this can compromise plant fertility. This was illustrated by Wijnker *et al*. (2012) who induced complete CO suppression through the RNA interference (RNAi)‐mediated knock‐down of the essential meiotic recombinase DISRUPTED MEIOTIC cDNA 1 (DMC1) in an F_1_ of *A. thaliana* (Wijnker *et al*., 2012). The knock‐down of *DMC1* caused non‐recombinant (univalent) chromosomes to randomly segregate to gametes, leading to high aneuploidy through the mis‐segregation of chromosomes. Consequently, balanced gametes were only formed at a low incidence in *A. thaliana* (about 3%), but this frequency would be much lower in other species when more chromosome pairs segregate randomly (Dirks *et al*., 2009). Therefore, complete CO suppression will present a strong bottleneck for the generation of reverse‐breeding offspring, especially in species with a high chromosome number, including various crops. A possible alternative may lie in reducing CO formation rather than completely suppressing it (Dirks *et al*., 2009). Reducing CO formation will still lead to the recovery of spores without CO events (CSLs), but also to spores with low CO numbers (Figure 1). This would not only be useful for the generation of CSLs through backcross schemes but also benefits reverse breeding.

The recreation of a heterozygous genotype (hereafter referred to as a full hybrid) is only possible if the two reverse‐breeding offspring that form the parental pair (i.e. two CSLs) have perfectly complementing genotypes (Figure 1a). If one of the two genotypes in the pair experienced a CO the resulting hybrid will be similar, but not genetically identical, to the full hybrid. For this reason, we refer to these plants as near‐full hybrids. As a result of a CO, near‐full hybrids will show a ‘decrease in heterozygosity’ (Figure 1a). This particular decrease of heterozygosity may not affect the phenotype of the near‐full hybrid, as compared with the full hybrid, however; it will only be detrimental if the particular regions that become homozygous affect hybrid performance (Huang *et al*., 2015). Therefore, it can be expected that several low‐recombinant offspring can be used as parental lines for hybrid breeding. Despite not knowing beforehand which homozygous regions affect hybrid performance, this can be determined experimentally by phenotyping a number of near‐full hybrids. If through incomplete CO suppression one can obtain parental lines that generate hybrids that are phenotypically identical to full hybrids, reverse breeding in species with higher chromosome numbers may become feasible.

Achieving the partial suppression of CO recombination in plants is possible because of the presence of two independent CO pathways (Higgins *et al*., 2004; Mercier *et al*., 2005). One of these is the ZMM protein pathway that, through the action of the heterodimer formed by MUTS HOMOLOG 4 and 5 (MSH4–MSH5) and other meiotic proteins, generates about 80–87% of the total number of COs, known as class‐I COs (Higgins *et al*., 2004; Mercier *et al*., 2005). The ZMM proteins are required during prophase I for the stabilization of recombination intermediates, and the disruption of this pathway by the loss of function of proteins like MSH4 or MSH5 results in the elimination of class‐I COs. Indeed, *msh4* and *msh5* mutants show on average 1.2 and 1.6 chiasmata per cell during meiosis, respectively, versus the nine to 10 chiasmata formed in wild‐type meiosis (Higgins *et al*., 2004, 2008; Wijnker *et al*., 2019). Therefore, in the event that either MSH4 or MSH5 is non‐functional, recombinant and non‐recombinant chromosomes will segregate at meiosis and both viable and non‐viable gametes will be formed (Higgins *et al*., 2004; Lu *et al*., 2008).

### VIGS to downregulate meiotic genes in *A. thaliana*

In its former design, reverse breeding was achieved by the RNAi‐mediated suppression of CO formation, which required the presence of a stable transgene in the genome of the heterozygote used to generate CSLs (Wijnker *et al*., 2012). For this transgene to be present in the heterozygote, one of the parental lines was stably transformed and used in a cross to give rise to an achiasmatic hybrid. When reverse breeding is to be applied to heterozygotes chosen from outcrossing populations, this approach would be unfeasible because no parental lines are available. To overcome this problem, meiotic recombination can be suppressed by using a transient silencing technique like VIGS, in which a plant is inoculated with a modified virus carrying a plant sequence that causes the silencing of the target gene of interest. Thanks to the development of a large repertoire of viral systems, VIGS has been routinely exploited in several plant species to modify the expression of genes involved in a wide number of processes, including meiosis (Senthil‐Kumar and Mysore, 2011b; Bennypaul *et al*., 2012; Bhullar *et al*., 2014). One of the most commonly used VIGS systems is based on the positive single‐stranded RNA tobacco rattle virus (TRV), which has been effectively applied in *Zea mays* (maize), *Papaver somniferum* (poppy), *Solanum *spp., *Nicotiana tabacum* (tobacco) and *A. thaliana* (Liu *et al*., 2002a,b; Brigneti *et al*., 2004; Hileman *et al*., 2005; Burch‐Smith *et al*., 2006; Senthil‐Kumar and Mysore, 2011b; Zhang *et al*., 2013).

The TRV‐VIGS system consists of a bipartite viral genome encoded on two vectors: TRV1 and TRV2. TRV1 is necessary for the replication and movement of the virus whereas TRV2 encodes the coat protein and other non‐structural proteins and harbours a cloned fragment of the plant target sequence (Ratcliff *et al*., 2001; Burch‐Smith *et al*., 2004). *Agrobacterium tumefaciens*‐mediated co‐inoculation followed by plant cell transformation and co‐expression of the TRV1 and TRV2 vectors in the plant leads to the formation of an active RNA virus. The viral replication process generates double‐stranded RNA (dsRNA) molecules that trigger an immune response through the activation of the RNA‐induced silencing complex (RISC) (Ruiz *et al*., 1998; Hamilton and Baulcombe, 1999; Baulcombe, 2004). The dsRNAs generated are initially processed into small‐interfering RNA (siRNA) molecules and then loaded into RISC. Sequences homologous to the siRNAs will be recognized as components of the viral genome, which ultimately causes the cleavage of both the viral as well as the endogenous mRNA target (Robertson, 2004; Kalantidis *et al*., 2008; Unver and Budak, 2009; Becker and Lange, 2010).

We tested whether COs can be efficiently reduced using TRV‐VIGS to silence *MSH5* in a wild‐type F_1_ *A. thaliana* hybrid. As TRV transmission to the offspring has been reported in *Solanum lycopersicum* (tomato) and *Nicotiana benthamiana* in low rates (10–15%), but not in *A. thaliana* (Senthil‐Kumar and Mysore, 2011a), VIGS‐mediated transient suppression of CO formation would allow the recovery of fully fertile, transgene‐free DH offspring from a reverse‐breeding experiment. These offspring will result from gametes carrying non‐recombinant (CSLs) and low‐recombinant chromosomes. In addition, we tested whether the DH lines generated could be used to: (i) restore the starting hybrid genotype and/or phenotype; and (ii) generate a population of near‐full hybrids in which we could experimentally test how hybrid performance is affected by the presence of certain homozygous regions across the genome.

## RESULTS

### Testing VIGS to modify meiosis

We first asked whether TRV could potentially target genes expressed in floral tissues and whether TRV affects plant fertility. To this end we exploited a commonly used positive control for VIGS experiments: a TRV vector targeting the gene *PHYTOENE DESATURASE* (*PDS*). The PDS protein is required in the chlorophyll biosynthesis pathway and its silencing results in strong photobleaching of plant tissues (Burch‐Smith *et al*., 2006). Four 3‐week‐old Col‐0 wild‐type plants were inoculated with TRV‐*PDS* and about 10–12 days after inoculation we observed the incipient signs of photobleaching in the developing young rosette leaves. White flower buds developed on all inoculated plants about 4 weeks after inoculation (Figure S1). These results suggest the possibility to target genes in meiotic tissues in *A. thaliana* using VIGS.

Previous studies that used the TRV‐VIGS system have reported mild or the absence of disease symptoms in *A. thaliana* (Burch‐Smith *et al*., 2006). Nonetheless, we decided to test whether TRV itself might compromise plant fertility and could affect silique elongation: phenotypes that could be mistaken for meiotic CO mutant phenotypes in *A. thaliana*. To this end we inoculated five Col‐0 plants with TRV containing an inactive short fragment of the *GUS* reporter gene system (Tameling and Baulcombe, 2007; Lu *et al*., 2008; Wu *et al*., 2011). We then checked whether TRV could be detected by reverse transcription polymerase chain reaction (RT‐PCR) in flower buds of Col‐0::TRV‐*GUS* plants. For this purpose we designed primers to detect the RNA polymerase encoded by TRV1. RT‐PCR confirmed the presence of TRV1 in Col‐0::TRV‐*GUS*, whereas no obvious amplicons were found in Col‐0 wild‐type control samples grown under the same conditions (Figure S2).

We then evaluated silique development in Col‐0::TRV‐*GUS* plants and observed that short siliques were not formed on TRV‐*GUS*‐treated plants (Figure S1). For certainty, we checked whether the viable seed set was reduced in elongated siliques. We counted the number of seeds in 10 random siliques from the main and lateral branches in three of these plants (Data S1). The lowest numbers of viable seeds in siliques of Col‐0::TRV‐*GUS* were 42, 45 and 46, which were never lower than the lowest viable seed set (22 seeds) found in Col‐0 control plants (Data S1). We therefore concluded that TRV does not affect plant fertility. For this reason, we used non‐inoculated plants (instead of Col‐0::TRV‐*GUS*) as controls in further experiments.

### VIGS‐mediated downregulation of *MSH5* causes semi‐sterility

Mutants of *mutS homolog 5* (*msh5*) are semi‐sterile and we therefore expected the VIGS‐mediated silencing of *MSH5* to result in a low seed set in treated plants (Lu *et al*., 2008). To test this, we generated a TRV2 vector carrying a sequence identical to a 242‐bp fragment of *A. thaliana MSH5*, spanning exon 4 to exon 7, and common to the three annotated splicing variants of *MSH5* found in https://plants.ensembl.org/index.html. The fragment was amplified from cDNA and cloned into TRV2 and confirmed by Sanger sequencing. We then used the TRV‐*MSH5* construct generated to inoculate four 3‐week old Col‐0 wild‐type plants.

To test for the presence of TRV in floral tissues, we assessed TRV1 expression through RT‐PCR in flower buds of treated and non‐treated plants. We confirmed TRV1 expression in treated plants but TRV1 could not be detected in control plants (Figure S2). To test for the presence of active TRV2, we designed primers that could amplify the *MSH5* region cloned into TRV*‐MSH5* and performed quantitative RT‐PCR (qRT‐PCR). For comparison, we used two reference genes *At4G26410* and *At3G47060* (see Experimental procedures). These analyses showed that *MSH5* expression in treated plants was 100–900‐fold higher compared with wild‐type controls. The high levels of *MSH5* expression in treated plants can be logically explained by the presence of the active TRV‐*MSH5* virus in flower buds (Liu *et al*., 2002a,b), whereas the low *MSH5* levels detected in controls corresponds to the expression of endogenous *MSH5* (Figure S2).

To test whether TRV‐*MSH5* could induce a reduction of endogenous *MSH5* transcripts, we designed primers binding outside the *MSH5* region cloned into TRV2, and checked the *MSH5* expression level in Col‐0::TRV‐*MSH5* and Col‐0 wild‐type controls. As *MSH5* expression is restricted to meiocytes (Lu *et al*., 2008), we also assessed in our samples the relative expression of another meiotic gene, *REC8*, a meiosis‐specific α‐kleisin subunit of the cohesin complex (Cai *et al*., 2003). We found that the expression levels of both *MSH5* and *REC8* showed a large variation of approximately two‐ to threefold among the untreated control samples when either of the two reference genes was used. This high variation hinders the possibility to faithfully compare *MSH5* and *REC8* expression between treated and untreated samples. Nonetheless, we did observe that the highest *MSH5* expression in treated plants was never higher than the lowest *MSH5* expression in Col‐0 controls (Figure S2). High variability in *MSH5* expression in control samples prohibits the clear quantification of the extent to which *MSH5* expression is downregulated in treated plants, however.

**Figure 2:**
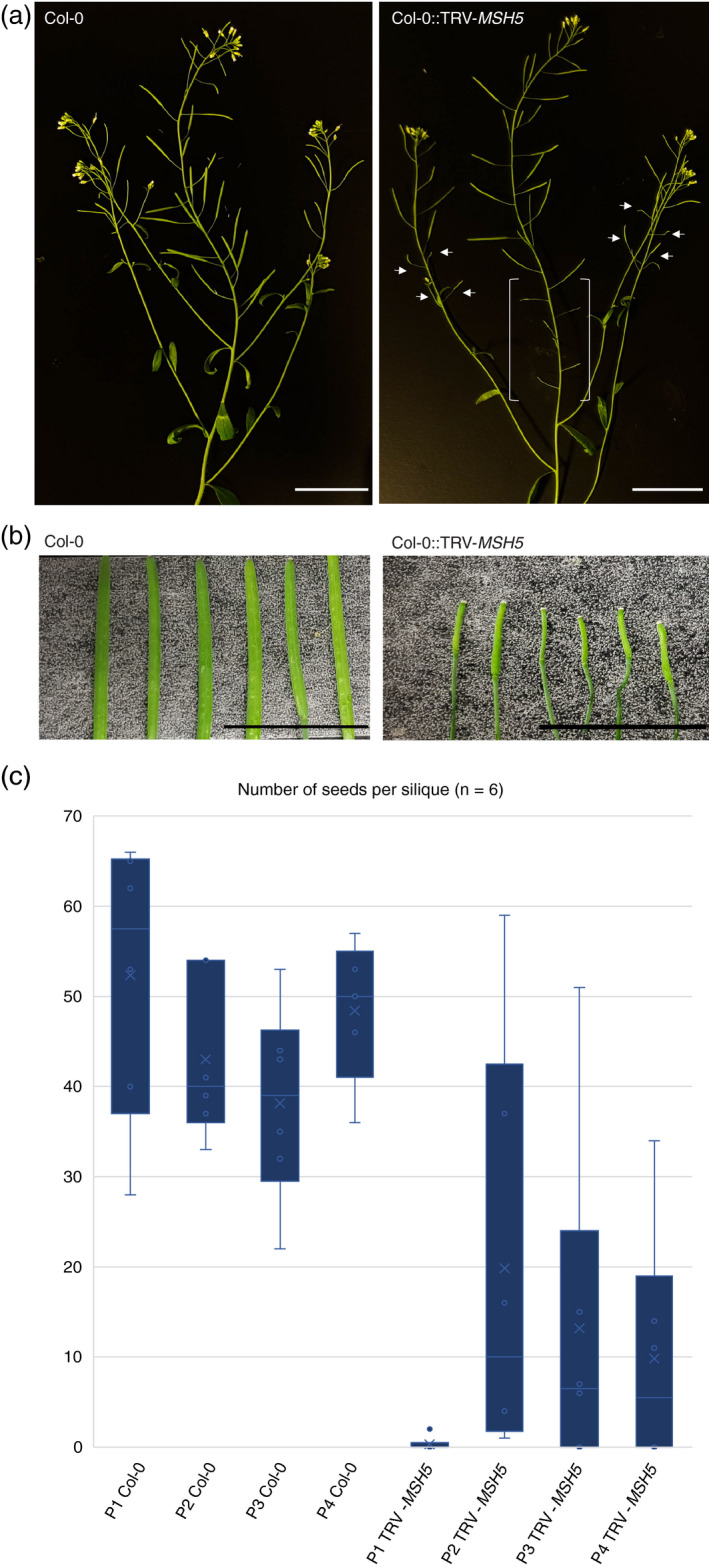
Treatment of plants with TRV‐*MSH5* leads to the formation of short siliques on main and lateral branches of the inflorescence. (a) Col‐0 wild‐type plants show long siliques, whereas several short siliques are present (indicated with white arrows and brackets) in TRV‐*MSH5*‐treated plants (scale bar: 3 cm). (b) Comparison of the first six siliques developed on the main stem of the inflorescence in Col‐0 and Col‐0::TRV‐*MSH5* (scale bar: 13 mm). (c) Number of viable seeds found in the first six siliques of four plants treated with TRV‐*MSH5* and four Col‐0 controls.

To assess the impact on fertility phenotypically in plants treated with TRV‐*MSH5*, we compared the silique lengths of Col‐0::TRV‐*MSH5* and control plants. We observed that the treated plants displayed a number of very short siliques at the base of the main inflorescence, whereas non‐inoculated Col‐0 wild‐type controls showed long, well‐elongated siliques (Figure 2a,b). Also, the first developing siliques on the lateral branches were very short in treated plants, contrasting with the long siliques found on control plants (Figure 2a). These results indicate that growth conditions did not cause the semi‐sterile phenotype observed in treated plants and that Col‐0::TRV‐*MSH5* plants clearly display a reduction of silique length in comparison with control lines.

To determine to what extent this reduced length represented a decrease in the seed set of treated plants, we quantified the number of viable seeds in the first six siliques on the main inflorescence in treated and control plants. The median number of seeds per silique was 44 in control plants (average = 45, SD = 12) whereas TRV‐*MSH5*‐treated plants had a median value of two seeds per silique (average = 11, SD = 17), highlighting a significant reduction in seed set (Student’s *t*‐test, α = 0.05; *P* < 0.01; Figure 2b,c; Data S1). Seed set was highly variable both within as well as between four TRV‐*MSH5*‐treated plants: the most extreme reduction was to an average of 0.3 seeds per silique (SD = 0.7) in plant P1, but ranged to an average of 19.8 (SD = 21) seeds per silique in the first six siliques in plant P2 (Figure 2c; Data S1). We concluded that TRV‐*MSH5* can induce a strong reduction in seed set, similar as described for *msh5* mutants (Lu *et al*., 2008), but phenotypic variation exists within and among treated plants.

**Figure 3:**
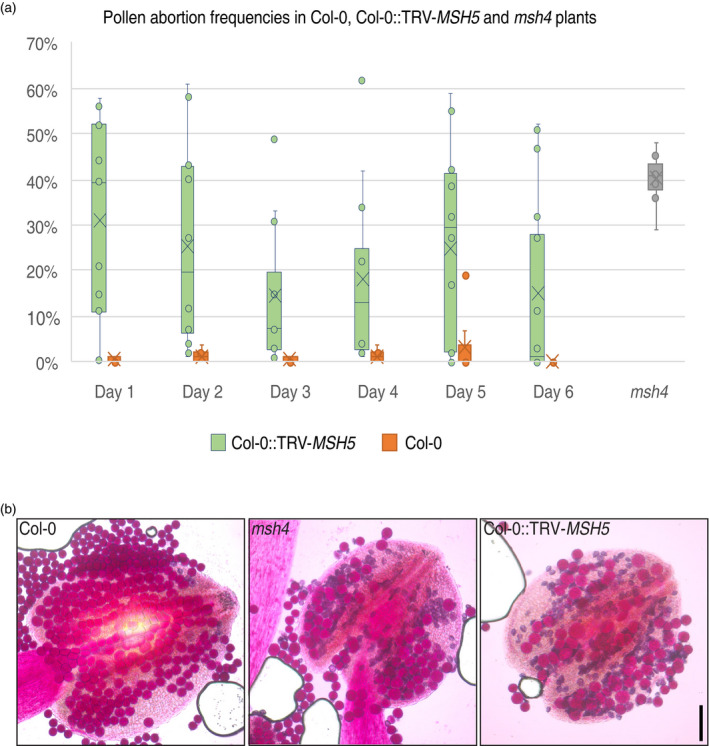
Pollen abortion in Col‐0 wild‐type, Col‐0::TRV‐*MSH5* and *msh4* plants. (a) The graph represents differences in pollen abortion rates during six consecutive days between flowers of four Col‐0 controls (*n* = 47), four Col‐0::TRV‐*MSH5* plants (*n* = 80) plus three *msh4* plants (*n* = 9), used for comparison. (b) Images of pollen stained with Alexander’s staining solution show viable pollen (magenta) produced by a Col‐0 wild‐type plant, whereas high numbers of aborted pollen (blue) are produced by *msh4* and Col‐0::TRV‐*MSH5* plants (scale bar: 1 mm).

The random segregation of univalent chromosomes in *msh5* mutants leads to aneuploidy among gametes and causes pollen abortion. We therefore characterized pollen abortion rates in four TRV‐*MSH5*‐treated Col‐0 plants and four non‐treated Col‐0 control plants. The number of dead pollen per 100 pollen grains was recorded in flowers on the main inflorescence for six consecutive days, starting from the moment in which the first flowers opened (32 days after inoculation) (Figure 3). Pollen abortion in control plants averaged at 1.3 per 100 pollen (1.3%), and was consistently below 5% in all but two of the flowers examined (*n* = 47, SD = 2.9; Figure 3; Data S1). By contrast, the average pollen abortion in Col‐0::TRV‐*MSH5* plants was higher at 21.9 per 100 pollen measured over all flowers (*n* = 80, SD = 20.9; Figure 3; Data S1). The comparison of pollen abortion distributions between treated and wild‐type plants indicates that three out of four treated plants show a significantly higher pollen abortion rate than control plants (Kolmogorov–Smirnov test, one sided, alpha = 0.05).

To test whether such rates were comparable with mutants with a dysfunctional class‐I CO pathway, we used *msh4*−/− mutant plants as a control. The *msh4* mutants had an average abortion rate of 40.3% and was rather constant, with a small standard deviation of 5.4 (*n* = 9 flowers) (Figure 3; Data S1). Pollen abortion rates in the flowers of treated plants was on average lower, with higher variation. Of 80 flowers that were counted over the first 6 days of flowering, 74% showed pollen abortion rates of lower than 40.3%, and 26.0% showed a higher pollen abortion rate. Two plants had low averages of pollen abortion of 6.0% (SD = 11.6) and 8.3% (SD = 11.6), of which only one (out of seven) and five (out of 25) flowers showed more than 5% pollen abortion, respectively. Two plants showed higher levels of pollen abortion of 32.8% (SD = 19.2) and 30.4% (SD = 19.1), in which 17 (out of 18) and 27 (out of 30) flowers showed pollen abortion above 5% (Figure 3; Data S1). Our results demonstrate that TRV‐*MSH5* induces pollen abortion in treated plants, but the silencing phenotype is very variable. As for our seed set data, we observed that there is considerable variation between plants and between flowers within treated plants, suggesting that the silencing effect of TRV‐*MSH5* is not fully penetrant.

### TRV‐*MSH5* induces univalent segregation during meiosis in *A*.* thaliana* F_1_ hybrids

The cause of semi‐sterility in *msh5*−/− is the loss of class‐I COs and the random segregation of univalent chromosomes at the first meiotic division. To assess directly whether the observed semi‐sterility in our experiments has a similar cause, one would expect to observe a meiotic phenotype in TRV‐*MSH5* as described for the *msh5* mutant. In *msh5*−/−, univalents and bivalents are present from late diplotene to metaphase I. The visible consequences of univalent segregation in later meiotic stages are the appearance of unbalanced chromosome numbers in daughter cells and polyad formation (Lu *et al*., 2008). As the phenotypic effects (i.e. reduced silique elongation and high pollen abortion) of inoculation with TRV‐*MSH5* were strongest in the first opening flowers, we sampled the first developing flower buds in a subsequent experiment for the generation of meiotic cell spreads. As meiosis precedes flower opening by approximately 7–9 days in *A*.* thaliana* (Liu *et al*., 2018), we sampled flower material 8 days before the anticipated opening of the first flowers (i.e. 3 weeks post‐inoculation). Flower buds were cut from developing rosettes of TRV‐*MSH5*‐treated F_1_ (Landsberg *erecta* × Columbia‐0, hereafter L*er* and Col‐0, respectively) hybrid plants and buds of appropriate size (0.6 mm) were selected for slide preparation.

**Figure 4:**
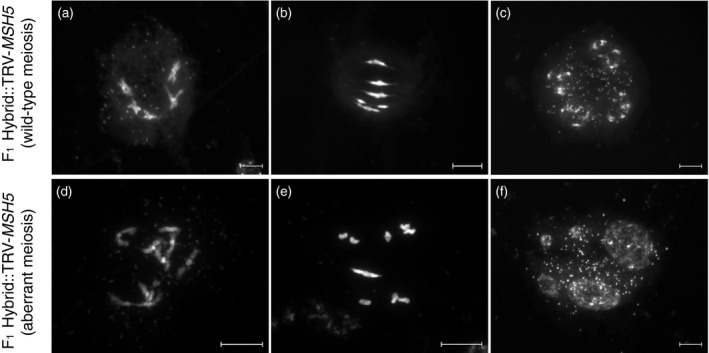
Meiotic cell complements of TRV‐*MSH5*‐treated F_1_ hybrid plants show phenotypes consistent with reduced crossover (CO) formation. Phenotypes consistent with wild‐type meiosis (a–c) and aberrant meiosis (d–f). Meiotic progression in *Arabidopsis thaliana* wild‐type meiosis typically shows the presence of five bivalents at diakinesis (a) and metaphase I (b), as well as balanced tetrads with four similar‐sized nuclei (c). Comparable meiotic cell stages in different flower buds are consistent with reduced CO formation, with the presence of univalents at diakinesis and metaphase I (d and e, respectively) and polyads at the tetrad stage, in which more than four nuclei are present of irregular size (f). Scale bars: 10 μm.

We obtained 10 slides (each of one flower bud) in meiotic stages from late diplotene to tetrad stages and analysed chromosome segregation in 206 meiotic cells (Table 1). Seven slides showed meiotic stages consistent with wild‐type meiotic phenotypes: the presence of five bivalents at late prophase (late diplotene to metaphase I) and cells in later meiotic stages (anaphase I to tetrad) in which balanced chromosome numbers suggest the occurrence of regular disjunction at the preceding metaphase I. In two slides we observed distinctly different meiotic prophase phenotypes: meiotic cells show low numbers of bivalents per cell (0.6 bivalents on average, SD = 0.7, *n* = 17 metaphase‐I cells), and in all 38 late diplotene to metaphase‐I cells in which individual chromosomes could be observed we noted the presence of univalents (Table 1). Later meiotic stages on these slides, as well as cells in a third slide, showed aberrant chromosome numbers in daughter cells and the formation of polyads instead of tetrads after meiosis (Figure 4). These observations confirm that TRV‐*MSH5*‐treated plants show a phenotype similar to the *msh5* mutant: strongly reduced CO formation and univalent segregation resulting in unbalanced chromosome numbers in gametes that leads to semi‐sterility. The reported average bivalent frequency for *Atmsh5‐1* is 1.09 bivalents per cell (Higgins *et al*., 2008), which is higher but in the range of what we observed. We conclude that the poor seed set and low pollen viability observed in TRV‐*MSH5*‐inoculated plants probably results from unbalanced chromosome segregation during metaphase I.

**Table 1:** Quantification of bivalent frequencies and meiotic aberrations in meiotic cells from 10 different flower buds in TRV‐*MSH5*‐inoculated F_1_ hybrids

Sample	Number of bivalents						Cells with univalents	Anaphase I to tetrad stage		*n*
	5	4	3	2	1	0		Regular	Irregular	
L004	–	–	–	–	–	–	–	1	3	4
L011	18	–	–	–	–	–	–	4	–	22
L014	–	–	–	–	1	2	3	–	7	10
L027	11	–	–	–	–	–	–	5	–	16
L028	24	–	–	–	–	–	–	8	–	32
L030	9	–	–	–	–	–	–	8	–	17
L031	3	–	–	–	–	–	–	8	–	11
L032	35	–	–	–	–	–	–	2	–	37
L033	6	–	–	–	–	–	–	4	–	10
L034	–	–	–	3	4	8	35^a^	5	7	47

Each sample represents a slide with a spread of meiotic cells that stem from one flower bud. Slides were selected for showing meiotic stages ranging from late diplotene to tetrad stage. Bivalent numbers and the presence of univalents were quantified in cells at late diplotene, diakinesis and metaphase I. Regularity of chromosome segregation in later meiotic stages (from anaphase I to tetrad stage) was judged by the regular distribution of segregated chromosomes (from anaphase I to telophase) and nuclear size and number (in tetrad‐stage cells). Most slides show phenotypes exclusively consistent with wild‐type meiosis: the presence of five bivalents, no univalents and regular distribution of chromosomes during late meiotic stages. Slides L004, L014 and L034 show aberrant numbers of bivalents, high numbers of cells with univalents as well as later meiotic stages, with evidence of the mis‐segregation of chromosomes.

^a^ The number of ‘cells with univalents’ for slide L034 is higher than the summation of the numbers in the preceding columns. The reason for this is that we occasionally observed cells in late meiotic prophase (late diplotene) with clear univalents but in which the number of bivalents could not be determined.

### Downregulation of *MSH5* using VIGS changes the genetic composition of the offspring

To evaluate the feasibility of crossing with gametes resulting from the VIGS‐mediated reduction of recombination, we inoculated a total of 109 (52 + 42 +15) F_1_ L*er* × Col‐0 plants with TRV‐*MSH5* in three consecutive experiments to use in crosses. When plants started flowering, we tested the successful knock‐down of *MSH5* in F_1_ flowers before crossing by assessing pollen abortion rates in one of the anthers of each flower. We also tested pollen produced by three non‐inoculated plants, which remained viable throughout the crossing periods. Of these experiments we selected 27, 19 and 15 plants that showed high pollen abortion rates, indicative of a successful silencing of *MSH5* expression. Of these plants, 132, 77 and 60 flowers, respectively, were used to pollinate *GFP‐tailswap*, a haploid inducer line for *A*.* thaliana* (Ravi and Chan, 2010). The haploid offspring generated were left for self‐fertilization to give rise to 111 diploid DH offspring that were then genotyped for 42 kompetitive allele‐specific PCR (KASP) markers (Semagn *et al*., 2014) evenly spaced over the genome (Figure S3; Data S2). Simultaneously, we generated a control population to assess the recombination rate in male meiosis by backcrossing wild‐type non‐inoculated F_1_ hybrids to a *male sterile 1* (*ms1*−/−) mutant of L*er* (hereafter we refer to this population as BC1).

**Figure 5:**
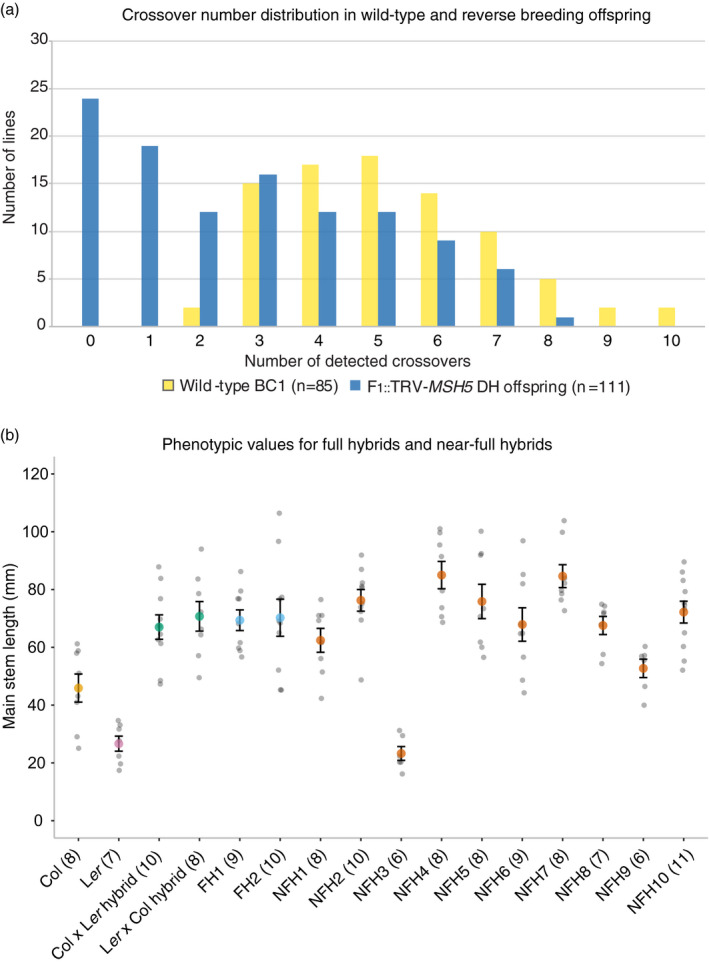
Crossover (CO) distributions in reverse‐breeding offspring and wild‐type BC1 offspring (a) and comparison of hybrid phenotypes (b). (a) Observed CO number in wild‐type BC1 offspring (in yellow) and reverse‐breeding doubled haploid (DH) offspring (in blue). Note that reverse‐breeding DH offspring are enriched for DHs, having 0 and 1 COs. (b) Hybrid phenotypes for main stem length at the moment of flowering of parental lines, reciprocal F_1_ hybrids, full hybrids (FH) and near‐full hybrids (NFH). Parental lines Col‐0 and L*er* (averages shown in yellow and pink, respectively), Col‐0 × L*er* reciprocal hybrids (green), full hybrids (blue) and near‐full hybrids (orange). Error bars represent standard error of the mean. Genotypes of near‐full hybrids are presented in Data S2.

Among the 111 DH offspring, we identified 24 DHs (20 different genotypes) with no detectable recombinant chromosomes (Data S2). These lines most likely carry non‐recombinant chromosomes, but with our marker set we cannot exclude the possibility of distal COs having occurred on chromosomes. These lines are henceforth referred to as DH_0_ to differentiate these offspring from other DHs (with detectable CO) in the following section. The population is significantly enriched for DH_0_ lines (i.e. derived from gametes without detectable COs) when compared with our BC1 control population of 85 plants (Kolmogorov–Smirnov test, α = 0.01; Figure 5a). Among these 20 DH_0_ genotypes we identified six complementing parental pairs that, when crossed, would recreate the starting hybrid (Data S2). All DH offspring in our population developed normally and were fully fertile. This shows that VIGS can transiently modify meiotic recombination in a wild‐type hybrid and change the genetic make‐up of the offspring derived from that hybrid.

### Recreation of hybrid genotypes and phenotypes using reverse‐breeding offspring

For the exact recreation of the heterozygous genotype a complementary DH_0_ pair is required, but in breeding practice the recreation of the hybrid phenotype will be the ultimate goal. The use of a DH_1_ (i.e. a DH with one detected recombinant chromosome) in a cross to recreate a hybrid leads to a decrease of heterozygosity (DOH) in the reconstituted hybrid distal to the CO position (Figure 1). We hypothesized that only a DOH that negatively affects the hybrid phenotype is of concern for reverse breeding. In our offspring we identified 19 DH_1_s and 12 DH_2_s, with one and two detected COs per genome, respectively, with the remainder of 56 DHs having between three and eight COs detected, which is in the range of wild‐type meiosis and probably results from the incomplete penetrance of VIGS (Data S2). Depending on CO positions in the DH_1_ and DH_2_ offspring, we noted the possibility of identifying four additional parental pairs (of DH_0_ with DH_1_) in which a near‐full hybrid would show a DOH that was less than 2.5% of the total genome length. Seven parental pairs would give rise to near‐full hybrids in which DOH is <5%. In one parental pair (DH_1_ line 44 × DH_2_ line 41) a DOH due to a CO on chromosome 1 in one parental line was partly compensated for by a CO event on the same chromosome in the other parent. This is similar to the DH_1_ × DH_2_ cross that was illustrated in Figure 1(a) and generated a near‐full hybrid with a DOH of 4.17% (Data S2).

The phenotypic impact of DOH can be explored experimentally. We therefore crossed different DH lines to create near‐full hybrids with increasing levels of DOH ranging from 1.28 to 32.07% (Data S2). These were grown together with the starting heterozygote and full hybrids (recreated heterozygotes), and then standard growth parameters were compared: flowering time (FT), main stem length (MSL), rosette diameter (RD) and dry weight (DW) at flowering time. No significant differences were found between the starting hybrid and the two recreated full hybrids (one‐way analysis of variance, ANOVA: FT, *P* = 0.3015; MSL, *P* = 0.9347; RD, *P* = 0.8655; DW, *P* = 0.2697; Figures 5 and S4). Also, no significant differences between the full hybrid and the near‐full hybrids were found, with the exception of one: a near‐full hybrid (NFH3) that has a similar short stem length as one of its parental lines (Figure 5b), which is likely to be caused by homozygosity of the main effect *erecta* locus that is homozygous in this specific hybrid (Stinchcombe *et al*., 2009). Interestingly, hybrids with the highest DOH (i.e. NFH4, NFH5, NFH7 and NFH10 with DOH of 32.07, 28,08, 31,03 and 21.46 respectively) do not display a reduced main stem length (Figure 5b, Data S2).

## DISCUSSION

Here we described that VIGS can be used to downregulate the expression of a meiotic gene (*MSH5*) in *A*.* thaliana*. The *A*.* thaliana msh5* mutant shows a desynaptic phenotype in which mainly univalent chromosome pairs segregate during the first meiotic division, leading to reduced fertility. TRV‐*MSH5*‐treated plants showed a similar semi‐sterile phenotype with a strongly reduced seed set, high levels of pollen abortion in flowers, the appearance of univalents after meiotic prophase and the presence of unbalanced chromosome numbers in meiotic spores. It was evident, however, that the semi‐sterility phenotype showed great variation between flowers in the same plant, and also between plants, consistent with the incomplete penetrance of VIGS silencing in *A*.* thaliana* reported previously (Burch‐Smith *et al*., 2006). In spite of this incomplete penetrance, we were able to generate offspring from TRV‐*MSH5*‐inoculated hybrid plants that showed no COs or were significantly reduced in CO numbers.

Control experiments detecting TRV1 and TRV2 expression through RT‐PCR and qRT‐PCR, respectively, on flower buds showed that TRV‐*MSH5* was strongly expressed in treated plants. Only TRV‐*MSH5* induced a semi‐sterile phenotype as plants expressing TRV‐*GUS* neither developed short siliques nor produced low numbers of viable seeds. Testing whether TRV‐*MSH5* expression also induced a corresponding decrease in endogenous *MSH5* was not successful, however. There may be several reasons for this. Throughout, we have shown that the penetrance of an *MSH5*‐silencing phenotype is incomplete and highly variable. The inflorescences tested are likely to have contained both silenced and non‐silenced cells, and possibly the non‐silenced cells introduce significant variation that preclude us from accurately assessing the downregulation of *MSH5* by qRT‐PCR. The observation that silencing occurs mainly in the first developing flowers, deep within the developing rosette, further complicates the timing of harvest. Therefore, for more detailed analyses of the silencing of meiotic genes by VIGS, flower bud staging and the use of a visual marker coupled with viral expression (i.e. TRV‐*GFP*) (Tian *et al*., 2014) could be considered. Possibly, one might study the silencing of fluorophore‐tagged proteins directly in anthers using live imaging of meiosis (Prusicki *et al*., 2019).

In comparison with a previously published proof of concept for reverse breeding (Wijnker *et al*., 2012, 2014), the execution of reverse breeding is here greatly simplified and improved. The proof of concept made use of a specially designed transgenic hybrid expressing a dominant acting RNAi transgene to suppress CO formation. Consequently, five generations were required to obtain CSLs from a hybrid and six generations were required to complete reverse breeding: three to create a transgenic hybrid, and three to recreate it (Wijnker *et al*., 2014). By contrast, the current experiment required just two generations to generate CSLs from a hybrid plant and three generations in total to recreate a heterozygote as F_1_ seeds (Figure 1). Furthermore, all offspring recovered in this experiment are transgene‐free and fertile, whereas in the previous set‐up half of the offspring were transgenic and semi‐sterile, which implies a twofold increase in efficiency.

In our experiments we targeted *MSH5* rather than *DMC1*, as was done in the previous design, to reduce CO formation. Through this approach we obtained a DH population that was strongly enriched for plants in which we detected 0, 1 and 2 COs per genome. We obtained 20 of the possible 32 CSLs among our offspring, and we could use these lines to recreate the starting hybrid. Clearly, reverse breeding (i.e. obtaining complementing DH_0_) was feasible through partial CO suppression. As partial CO suppression also leads to the recovery of DH_1_ and DH_2_, we also wondered whether these could be used to recreate the starting hybrid. Crossing DH lines with CO events (i.e. DH_1_ and DH_2_) precluded the exact recreation of the genotype of the starting hybrid, but it allowed us to test whether a DOH would negatively impact the phenotype of the recreated F_1_. Our data suggest that a DOH will in some cases affect the F_1_ phenotype, but in other cases will not. This implies that DH_1_ and DH_2_ may be suitable breeding lines to recreate the hybrid phenotype following a reverse‐breeding experiment.

It is possible to estimate the expected DOH in near‐full hybrids resulting from a single CO. *Arabidopsis thaliana* has five linkage groups (chromosomes). One CO in the genome of a DH line recombines one linkage group (one‐fifth of the genome) and this CO exchanges anything between zero and 50% of the linkage group, which averages at one‐quarter of the linkage group (typically half a chromosome arm). The expected DOH caused by a single CO thus equals on average 1/4*1/5 (5%) of the total linkage map length. Of the ten near‐full hybrids (with one CO) that we could produce, five have a DOH of less than 5% in Mbp. This is exactly as predicted, as the *A*.* thaliana* genetic map correlates well with the physical chromosome length. The more chromosomes that a species has, the lower the relative DOH resulting from one CO. In a species with 10 chromosome pairs (e.g. maize), one CO on a chromosome causes a DOH of 2.5%. This decreases even further when, as in many species, COs are located relatively distally on chromosomes. Under such a scenario, not only DH_0_ but also DH_1_ and DH_2_ may prove worthy parental lines, provided that the resulting near‐full hybrids are phenotyped to assess their performance.

The complete experimental procedure showed the feasibility of using VIGS to transiently modify meiotic recombination in order to change the genetic composition of gametes. Two previous reports demonstrated the efficiency of a VIGS system (based on the barley stripe mosaic virus, BSMV) to reduce the expression of *DMC1* by 75–80% and *C‐Ph1* by 78.17% in *Triticum aestivum* (wheat) (Bennypaul *et al*., 2012; Bhullar *et al*., 2014). Having shown that, at least in *A*.* thaliana*, the modified composition of spores can give rise to offspring with altered characteristics opens up routes to also alter other meiotic processes, and use VIGS to increase recombination frequencies, for example (Fernandes *et al*., 2017).

The experiments in wheat suggest that VIGS‐mediated silencing of meiotic genes can be used to develop breeding strategies in other species too, although the most appropriate VIGS system will need to be considered for each particular case. For instance, VIGS based on BSMV has also been used successfully in *Secale cereale* (rye), *Brachypodium* spp., *Hordeum vulgare* (barley) and maize (Holzberg *et al*., 2002; Bruun‐Rasmussen *et al*., 2007; Pacak *et al*., 2010; Bennypaul *et al*., 2012; Groszyk *et al*., 2017), and VIGS based on turnip yellow mosaic virus (TYMV) has been exploited in *Brassica rapa* (Yu *et al*., 2018). VIGS to modify meiosis can be especially suitable for polyploids. Silencing efficiency is not influenced by gene copy number and it has even been proposed as a tool to downregulate entire gene families (Senthil‐Kumar and Mysore, 2011a; Fitzgerald *et al*., 2012). Indeed, several works have shown that VIGS can be efficiently applied in polyploids like wheat (Fitzgerald *et al*., 2012; Manmathan *et al*., 2013), *Solanum tuberosum* (potato; Brigneti *et al*., 2004; Faivre‐Rampant *et al*., 2004), *Brassica napus* (Álvarez‐Venegas *et al*., 2010) and *Gossypium barbadense* (cotton; Pang *et al*., 2013).

### Potential application of reverse breeding in other species

The feasibility of generating CSLs and the application of reverse breeding in other species should be evaluated in a case‐by‐case scenario. Apart from the VIGS system to be used, the chromosome number of the species may greatly impact the chances of obtaining viable offspring after reducing CO frequencies in breeding schemes. When CO suppression leads to the segregation of univalent pairs, the chance of recovering a balanced gamete is a direct function of the number of univalent pairs, and equals 1/2*^x^*, where *x* is the number of univalent chromosome pairs. Gamete viability exponentially decreases when univalent pairs increase. With this study, we have shown that CO suppression can be incomplete in order to increase the chance of obtaining gametes carrying non‐recombinant chromosomes only.

In a crop like maize (*x* = 10), the total absence of COs will yield a frequency of viable gametes that equals 1/2^10^ = 0.09%. Reported CO numbers in wild‐type maize vary from 20.5 to 38 per meiosis (Anderson *et al*., 2003; Li *et al*., 2015), so the suppression of class‐I COs, assuming an 87% decrease in COs, would result in three or six residual COs per meiosis and frequencies of viable gametes that equal 1/2^(10 − 3)^ (0.8%) and 1/2^(10 − 6)^ (6%), respectively. These frequencies represent an increase in fertility of between eight‐ and 66‐fold in comparison with complete CO suppression. Of the resulting gametes, 1/8th and 1/66th would directly generate CSLs if used for DH production.

The frequency of recovered reverse‐breeding offspring can also be heightened thanks to the development of more efficient haploid induction lines and/or tissue culture techniques. In recent years, haploid and DH production rates have increased in crops such as *Oryza sativa* (rice), barley, *Brassica rapa* or maize (Britt and Kuppu, 2016; Ishii *et al*., 2016; Kelliher *et al*., 2017; Naik *et al*., 2017; Ren *et al*., 2017; Yao *et al*., 2018; Jia *et al*., 2019). We anticipate that the development of transient silencing techniques for meiosis coupled with high rates of DH production will facilitate the application of reverse breeding‐like technologies in breeding set‐ups.

## Experimental procedures

### Plant material and growth

*Arabidopsis thaliana* plants used in crosses and for VIGS inoculation were grown in potting soil in growth chambers (Percival, https://www.percival‐scientific.com) with a 21/18°C and 16‐h light/8‐h dark cycle, with 50–60% relative humidity. Haploid offspring were grown under similar conditions in a glasshouse. For phenotyping, seeds of DH offspring, F_1_ hybrids, reconstituted full hybrids and near‐full hybrids were vernalized by sowing on wet filter paper and placing them in the dark at 4°C for 4 days to ensure uniform germination. Plants were grown on 4 cm × 4 cm rockwool blocks and watered with a flooding system with a Hyponex nutrient solution three times per week in a randomized block design with five blocks and two replicates per genotype in each block. The climate chamber conditions were set to a cycle of 16‐h light (125 µmol m^−2^ sec^−1^)/8‐h dark and 20/18°C, with 70% relative humidity. *Arabidopsis thaliana* accession numbers: L*er*‐1 (stock number CS76164; ABRC, https://abrc.osu.edu) and Col‐0 (stock number CS76113; ABRC).

### Plasmid construction and *Agrobacterium* inoculation

To generate TRV‐*MSH5*, we extracted RNA from Col‐0 flower buds and used this to synthesize cDNA using the RevertAid RT Kit (ThermoFisher Scientific, https://www.thermofisher.com). A region of 242 bp homologous to *MSH5* was amplified from this cDNA using primers to which *BamH*I (forward) and *Xba*I (reverse) restriction sites were added. The PCR product was obtained using the primer pair *MSH5*_Fw_, 5′‐CAGGATCCAAGCCATCGATCATTTACGC‐3′, and *MSH5*_Rw_, 5′‐CATCTAGAACTTGGACTTCACTGCCCAC‐3′. The PCR product was introduced into the vector TRV2 (pYL156) (Liu *et al*., 2002a,b) following a classical digestion–ligation reaction and verified by Sanger sequencing. After sequence verification, the TRV*‐MSH5* vector was transformed into *Agrobacterium tumefaciens GV3101* (*pMP90*) strain. The incubation and inoculation protocols were executed as described by Nimchuk *et al*. (2000). Plant inoculation was performed by leaf infiltration (Vaghchhipawala *et al*., 2011) with one of the VIGS vectors of choice: TRV2*‐MSH5*, TRV2*‐PDS* (stock reference CD3‐1047; ABRC) or TRV2‐*GUS* (Tameling and Baulcombe, 2007) in combination with TRV1 (pYL156, stock reference CD3‐1039; ABRC) in a 1:1 ratio.

### Expression analysis of TRV1, TRV2*‐MSH5* and endogenous *MSH5* in treated and non‐treated plants

Flower buds of Col‐0, Col‐0::TRV‐*GUS* and Col‐0::TRV‐*MSH5* plants grown under the same conditions were harvested in liquid nitrogen and stored at −80°C. RNA extraction was achieved using TRIzol and DNA synthesis was performed with the RevertAid RT Reverse Transcription Kit from ThermoFisher Scientific. Primers RT‐TRV1‐Fw, 5′‐CATGTTGGTGGGAAGAAGAGTGAACACAAG‐3′, and RT‐TRV1‐Rw, 5′‐GATTTGAATGAACCCAGGCGTATCTGCAG‐3′, were designed to detect the TRV1‐encoded polymerase by RT‐PCR.

The qRT‐PCR was performed using two reference genes: *At3G47060*, which encodes for a chloroplast‐localized FtsH protein and is stably expressed in the shoot apex (Sakamoto *et al*., 2003; Liu *et al*., 2010), and *At4G26410*, commonly used as a reference gene for different developmental stages and, in plants, subjected to different abiotic and biotic stress conditions (Hong *et al*., 2010; Kudo *et al*., 2016). The primers used to detect the expression of these two genes were qRT‐*At3G47060*‐Fw, 5′‐GGCTTGGTGCTCAACTTGAAGAG‐3′, qRT‐*At3G47060*‐Rw, 5′‐TGGTGCAACCACCATGCTTAAC‐3′, qRT‐*At4G26410*‐Fw, 5′‐GAGCTGAAGTGGCTTCCATGAC‐3′, and qRT‐*At4G26410*‐Rw, 5′‐GGTCCGACATACCCATGATCC‐3′, respectively. For assessing *REC8* and endogenous *MSH5* expression, the following primers were designed: qRT‐*REC8*‐Fw, 5′‐TCGTAGGGACGGATTTGCTGAG‐3′, qRT‐*REC8*‐Rw, 5′‐TGGTTGTGGTCTATCGTGTTCCTC‐3′, qRT‐*MSH5*‐Fw, 5′‐TGCTGAGCTATGGCCTTCAC‐3′, and qRT‐*MSH5*‐Rw 5′‐CCGCAAACTTGTCAACAGCA‐3′. To detect the expression of TRV2‐*MSH5* we used the following primers: qRT‐TRV2‐*MSH5*‐Fw, 5′‐GCACAGACTGGTATATCTTCGA‐3′, and qRT‐TRV2‐*MSH5*‐Rw, 5′‐GGTTTCTACAATTCGTTCGCTT‐3′.

### Pollen phenotyping

A total of four Col‐0 plants were inoculated with TRV‐*MSH5* 3 weeks post‐germination, and we checked the pollen of one or two anthers from a total of 80 flowers starting when the first flower opened (at 32 days post‐inoculation) (Data S1). Pollen from all the open flowers were sampled every day during six consecutive days and were stained using Alexander’s staining solution to observe pollen viability (Peterson *et al*., 2010). A total of 47 flowers of four Col‐0 non‐inoculated plants and nine flowers of four *msh4* plants grown at the same time and in the same tray were used as controls during the test period.

### Pollen phenotyping during crossing and DH production

Pollen of a total of 109 F_1_ hybrid plants of L*er* × Col‐0 inoculated with TRV‐*MSH5* was checked for up to 6 days and used in crosses in three consecutive experiments. Three non‐inoculated F_1_ hybrids were grown as wild‐type controls in each experiment as well as three or four plants that were inoculated with TRV‐*PDS* to silence *PDS* as a positive control (Burch‐Smith *et al*., 2006).

To produce DHs, the flowers of the F_1_ hybrid plants of L*er* × Col‐0 inoculated with TRV‐*MSH5* producing aborted pollen were used to pollinate the haploid inducer line *GFP‐tailswap* (Ravi and Chan, 2010). Haploid selection was performed as described by Wijnker *et al*. (2014). Among the 369 offspring produced we identified 113 haploid offspring, and for 111 of these we obtained DH seeds. To produce the 85 offspring used as the BC1 control population, non‐inoculated F_1_ hybrids grown under the same growing conditions were backcrossed to L*er ms1−/−* mutant plants. The BC1 offspring were also grown under the same growing conditions as the haploid population. Both subsets were genotyped for a total of 42 markers distributed over the whole genome (Figure S3; Data S2). Doubled haploids, in which elimination of the *GFP‐tailswap* genome was incomplete, were selected against based on plant phenotypes (i.e. aberrant growth of rosettes, flowers and seed set in the DHs). The absence of heterozygous genotype calls in offspring (doubled) haploids (i.e. for regions derived from the L*er* parent) further confirmed haploidy.

### Statistical analysis

The critical *D* value for the Kolmogorov–Smirnov test was calculated as *D* = *c*(α)√[(*n* + *m*)/(*nm*)], where *n* and *m* represent the different sample sizes.

### Phenotypical analysis of full hybrids and near‐full hybrids

At the moment of flowering, the FT was recorded and MSL, RD and DW were measured for each plant. Phenotypic data were corrected for spatial trends and block effects with the spats package in r, and the resulting spatial corrected raw data were used for further analysis. To test whether the crosses of two different combinations of DH_0_ resulted in phenotypes that differed from the two full hybrids (FH1 and FH2), comparisons were made between the parental and reciprocal wild‐type F_1_ using one‐way ANOVA. To assess the performance of the near‐full hybrids in comparison with the full hybrid, a Dunnett test was conducted in which FH2 was used as a control.

### Cytology

F_1_ hybrid flower buds were sampled at 18 days post‐inoculation. The inflorescences were incubated in Carnoy, a 3:1 mix of glacial acetic acid (HAc) and 99.8% EtOH, and kept overnight at 4°C. Inflorescences were then washed twice with 70% EtOH (in water) and stored at 4°C. Meiotic chromosome spreads were made as previously described in Ross *et al*. (1996), stained with 4′,6‐diamidino‐2‐phenylindole (DAPI) in Vectashield and analysed using a Zeiss microscope equipped with epifluorescence optics.

## AUTHOR CONTRIBUTIONS

EW conceptualized the research. JJBK, HDJ, AS and EW were involved in supervision and funding acquisition. VCB and EW planned the research and performed the crosses. NL helped with setting up VIGS experiments and with constructing the design. CY performed expression analyses. Cloning and VIGS experiments were performed by VCB. LH performed cytogenetic analyses with the help of EW. CBdS performed the genotyping. VCB, CLW, JJBK and EW designed, performed and analysed the phenotyping experiment. VCB and EW processed and interpreted the experimental data, designed the figures and drafted the article, with the help of AS and HdJ. All authors discussed the results and commented on the article.

## CONFLICT OF INTEREST

Rijk Zwaan BV holds a patent for reverse breeding. CBdS is a current employee of Rijk Zwaan and EW is a former employee. HdJ as well as JJBK previously received research funding from Rijk Zwaan BV.

## Supporting information


**Figure S1.** Positive and negative controls used in VIGS assays: Col‐0::TRV‐*PDS* and Col‐0::TRV‐*GUS.*(a) Col‐0 plants inoculated with TRV‐*PDS* display photobleaching affecting leaves, stem and flower buds at four weeks after inoculation. (b) Fully fertile Col‐0 plant inoculated with TRV‐*GUS*, used as a negative control (Scale bar 13 mm).

**Figure S2.** Expression analysis of TRV and *MSH5* in treated and control plants. (a) TRV1 expression was detected by RT‐PCR in Col‐0::TRV‐*GUS* and Col‐0::TRV‐*MSH5* plants but not in Col‐0 control plants. The line between Col‐0 controls and Col‐0::TRV‐*MSH5* samples indicates that these samples were run on different gels, but both sets of samples were generated and processed at the same time. (b) Increased expression of the *MSH5* gene fragmentpresent on TRV*‐MSH5* in Col‐0::TRV‐*MSH5* as compared to Col‐0 controls detected by qRT‐PCR. Note that the y‐axis is discontinuous. (c) qRT‐PCR analysis on endogenous *MSH5* expression in Col‐0::TRV‐*MSH5* and Col‐0 controls.

**Figure S3.** Physical positions of genetic markers used to genotype reverse breeding offspring. The names of used markers indicate the Col‐0 allele, the L*er* allele and the bp position in the Col‐0 reference genome.

**Figure S4.**
The phenotypes of parental lines, reciprocal F_1_ hybrids, full hybrids and partial hybrids. The three panels show the values corresponding to flowering time (a) in days afters sowing (DAS), rosette diameter (b) and dry weight (c). From left to right data are shown for the parental lines Col‐0 (average in yellow) and L*er* (pink), Col‐0 x L*er* reciprocal hybrids (green), full hybrids (FH, blue) and near‐full hybrids (NFH, orange). Error bars represent standard error of the mean. FH and NFH genotypes shown in Data S2.

**Data S1.** Seed set and pollen viability in controls and TRV‐*MSH5*‐inoculated Col‐0 plants.

**Data S2.** Genotypes of *Arabidopsis thaliana* reverse‐breeding offspring and BC1 control population. Genotypes and phenotypic values of the F_1_ hybrids, full hybrids and near‐full hybrids.

## Data Availability

All relevant data can be found within the article and its supporting materials.
