# Supplementary figures and images for: Meiotic crossover reduction by virus‐induced gene silencing enables the efficient generation of chromosome substitution lines and reverse breeding in Arabidopsis thaliana
Source: Plant J. 2020 Oct 20;104(5):1437–52. doi: 10.1111/tpj.14990 (PMC7756339; doi:10.1111/tpj.14990)

(a) Col-0::TRV-*PDS*

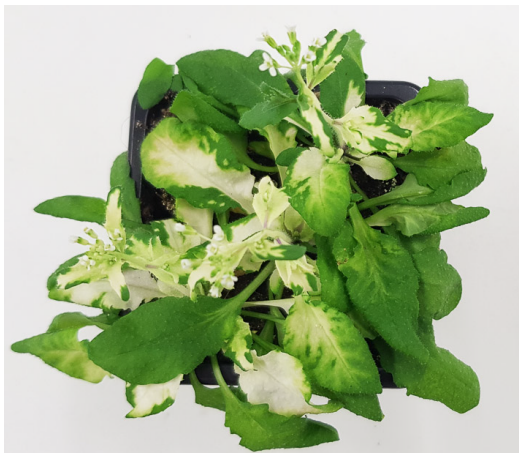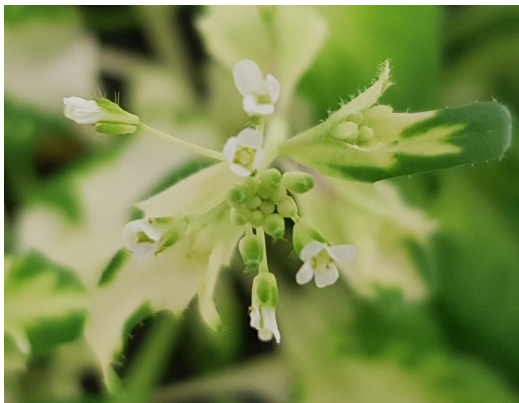

(b) Col-0::TRV-*GUS*

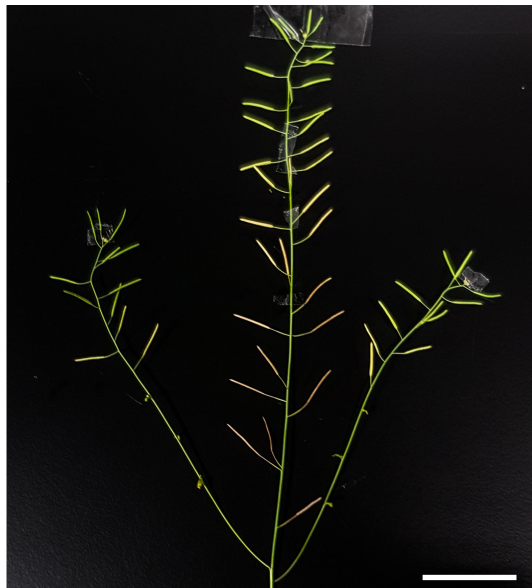

(c) Col-0::TRV-*GUS*

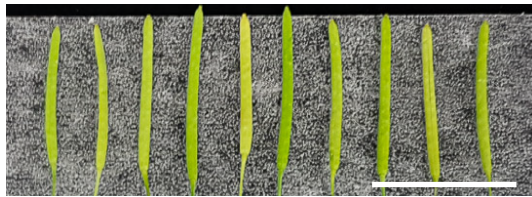

Supplement: Supplementary file 1 — Figure S1. Positive and negative controls used in VIGS assays: Col‐0::TRV‐PDS and Col‐0::TRV‐GUS.(a) Col‐0 plants inoculated with TRV‐PDS display photobleaching affecting leaves, stem and flower buds at four weeks after inoculation. (b) Fully fertile Col‐0 plant inoculated with TRV‐GUS, used as a negative control (Scale bar 13 mm). [file TPJ-104-1437-s001.pdf]

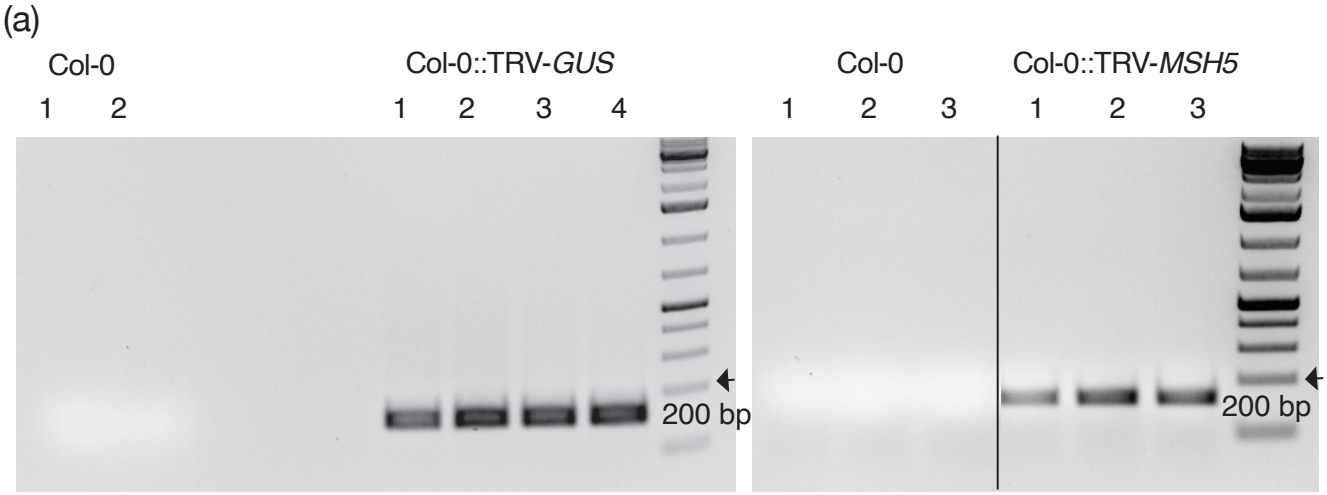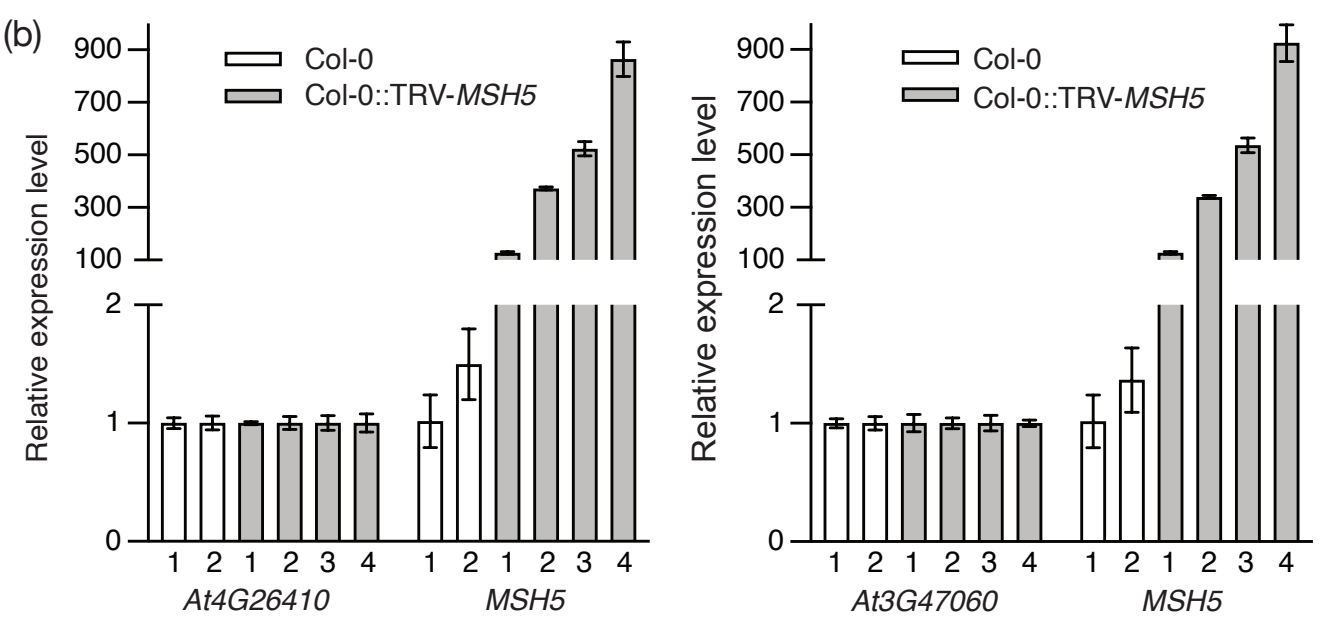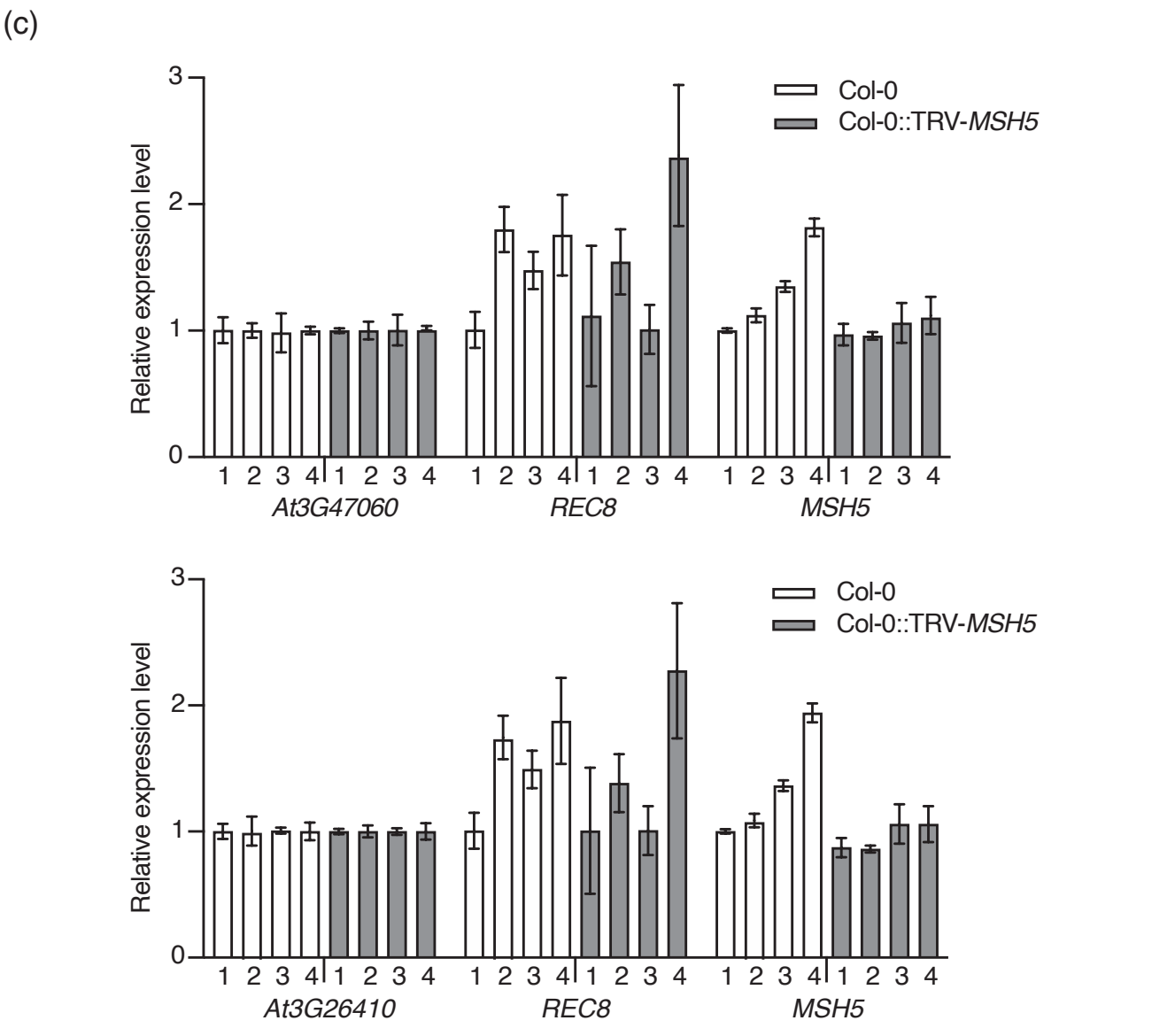

Supplement: Supplementary file 2 — Figure S2. Expression analysis of TRV and MSH5 in treated and control plants. (a) TRV1 expression was detected by RT‐PCR in Col‐0::TRV‐GUS and Col‐0::TRV‐MSH5 plants but not in Col‐0 control plants. The line between Col‐0 controls and Col‐0::TRV‐MSH5 samples indicates that these samples were run on different gels, but both sets of samples were generated and processed at the same time. (b) Increased expression of the MSH5 gene fragmentpresent on TRV‐MSH5 in Col‐0::TRV‐MSH5 as compared to Col‐0 controls detected by qRT‐PCR. Note that the y‐axis is discontinuous. (c) qRT‐PCR analysis on endogenous MSH5 expression in Col‐0::TRV‐MSH5 and Col‐0 controls. [file TPJ-104-1437-s002.pdf]

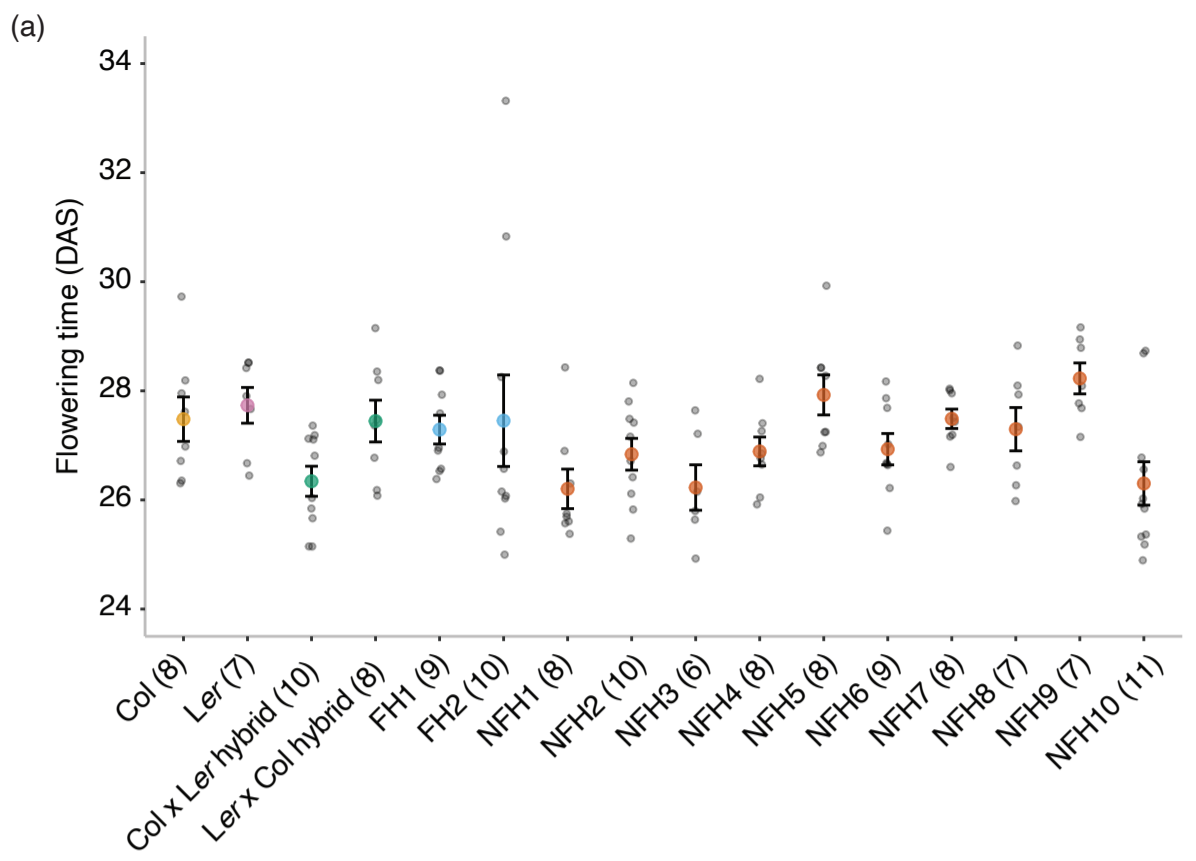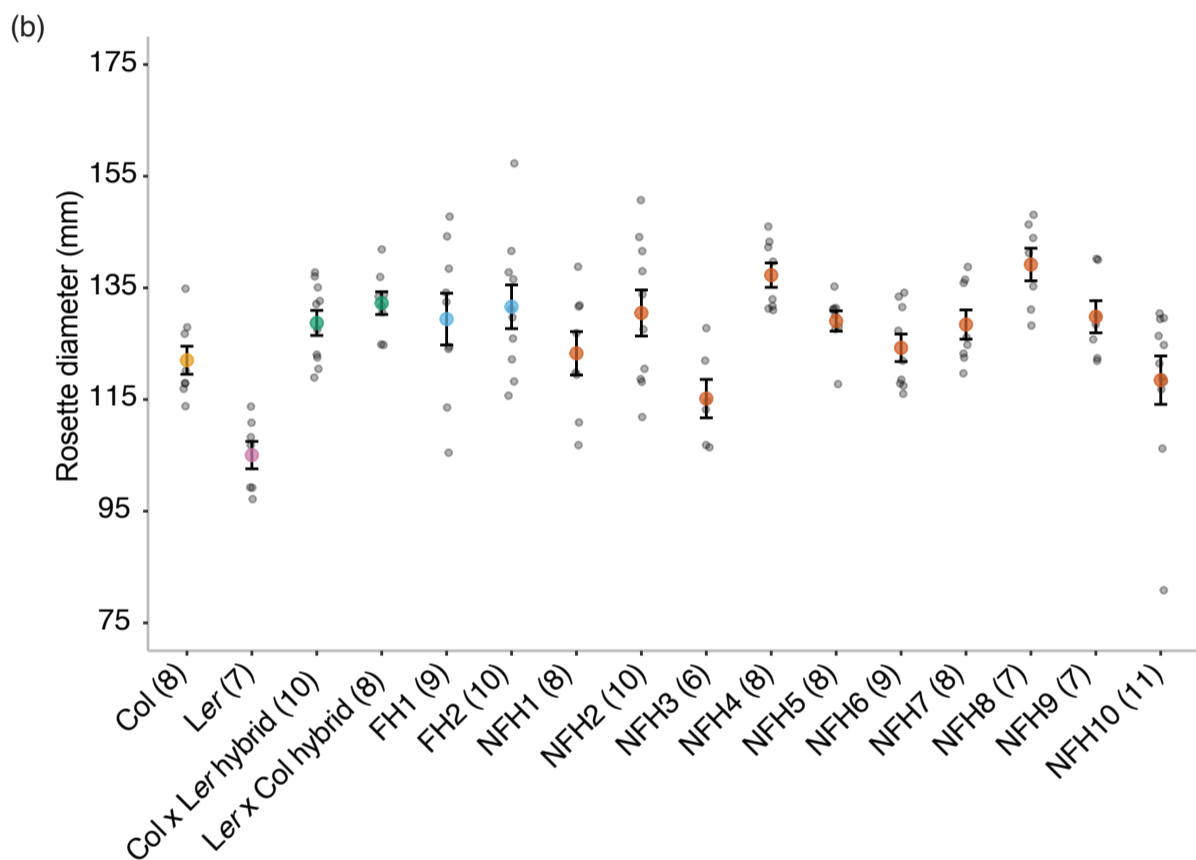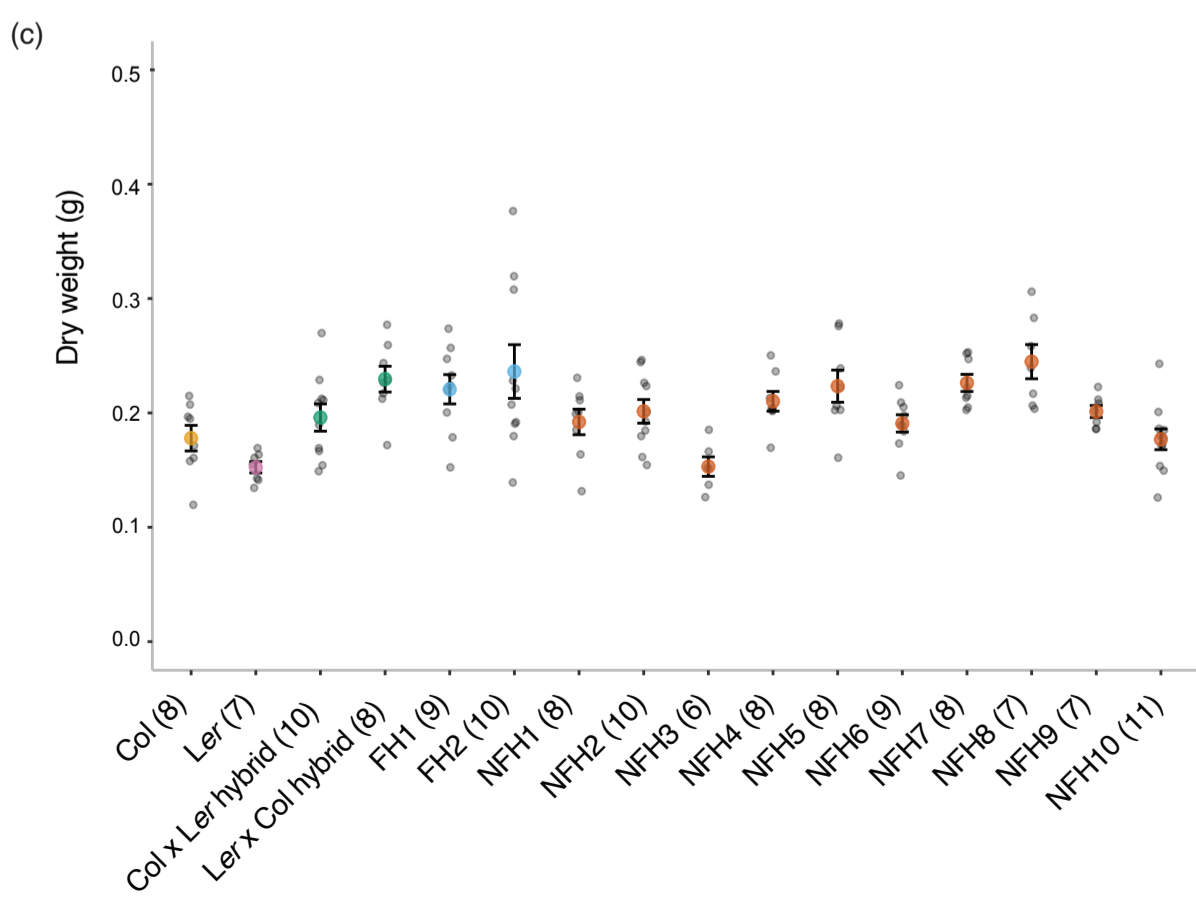

Supplement: Supplementary file 4 — Figure S4. The phenotypes of parental lines, reciprocal F1 hybrids, full hybrids and partial hybrids. The three panels show the values corresponding to flowering time (a) in days afters sowing (DAS), rosette diameter (b) and dry weight (c). From left to right data are shown for the parental lines Col‐0 (average in yellow) and Ler (pink), Col‐0 x Ler reciprocal hybrids (green), full hybrids (FH, blue) and near‐full hybrids (NFH, orange). Error bars represent standard error of the mean. FH and NFH genotypes shown in Data S2. [file TPJ-104-1437-s004.pdf]
