# Supplementary material for: Meiotic crossover reduction by virus‐induced gene silencing enables the efficient generation of chromosome substitution lines and reverse breeding in Arabidopsis thaliana
Source: Plant J. 2020 Oct 20;104(5):1437–52. doi: 10.1111/tpj.14990 (PMC7756339; doi:10.1111/tpj.14990)
Supplement: Supplementary file 7 [file TPJ-104-1437-s007.docx]

**Supporting materials legends**

Figure S1: Positive and negative controls used in VIGS assays: Col-0::TRV-*PDS* and Col-0::TRV-*GUS.* (a) Col-0 plants inoculated with TRV-*PDS* display photobleaching affecting leaves, stem and flower buds at four weeks after inoculation. (b) Fully fertile Col-0 plant inoculated with TRV-*GUS*, used as a negative control (Scale bar 13 mm).

Figure S2: Expression analysis of TRV and *MSH5* in treated and control plants. (a) TRV1 expression was detected by RT-PCR in Col-0::TRV-*GUS* and Col-0::TRV-*MSH5* plants but not in Col-0 control plants. The line between Col-0 controls and Col-0::TRV-*MSH5* samples indicates that these samples were run on different gels, but both sets of samples were generated and processed at the same time. (b) Increased expression of the *MSH5* gene fragment present on TRV*-MSH5* in Col-0::TRV-*MSH5* as compared to Col-0 controls detected by qRT-PCR. Note that the y-axis is discontinuous. (c) qRT-PCR analysis on endogenous *MSH5* expression in Col-0::TRV-*MSH5* and Col-0 controls.

Figure S3. Physical positions of genetic markers used to genotype reverse breeding offspring. The names of used markers indicate the Col-0 allele, the L*er* allele and the bp position in the Col-0 reference genome.

Figure S4. The phenotypes of parental lines, reciprocal F_1_ hybrids, full hybrids and partial hybrids. The three panels show the values corresponding to flowering time (a) in days afters sowing (DAS), rosette diameter (b) and dry weight (c). From left to right data are shown for the parental lines Col-0 (average in yellow) and L*er* (pink), Col-0 x L*er* reciprocal hybrids (green), full hybrids (FH, blue) and near-full hybrids (NFH, orange). Error bars represent standard error of the mean. FH and NFH genotypes shown in Data S2.
